# Diagnosis of the Diatom Community upon Biofilm Development on Stainless Steels in Natural Freshwater

**DOI:** 10.1155/2017/5052646

**Published:** 2017-05-25

**Authors:** Caroline Richard, Smita Mitbavkar, Jessem Landoulsi

**Affiliations:** ^1^Laboratoire de Mécanique et Rhéologie (LMR), EA 2640, Polytech'Tours, Université François Rabelais de Tours, 37200 Tours, France; ^2^CSIR-National Institute of Oceanography, Dona Paula, Goa 403 004, India; ^3^Sorbonne Universités, UPMC Univ Paris 06, 75005 Paris, France; ^4^CNRS, UMR 7197, Laboratoire de Réactivité de Surface, 75005 Paris, France

## Abstract

This paper reports the development of biofilms on stainless steels (SS) upon exposure in a natural freshwater ecosystem for about six months and focuses on the composition of diatom populations. By using environmental scanning electron microscopy (ESEM) technique, we provide a detailed description regarding diatom identification at species level as well as their main characteristics, including type, morphology, ability to form colony, and motility. Results reveal the presence of both prostrate (initial colonizers) and stalked (late colonizers) forms. Pennate diatoms,* Cocconeis placentula* and* Amphora coffeaeformis,* and a centric diatom,* Melosira varians*, are shown to be the abundant forms regardless of the SS type. Pennate diatoms dominate the community and are directly attached to the substratum, whereas the centric form is entangled in the biofilm matrix in a significant number. The dominance of adnate forms suggests that these cells are sturdy and successfully maintaining their population. In situ monitoring of the electrochemical response of immersed materials showed ennoblement of the open circuit potential, which seems to be due to the biogenic production of H_2_O_2_, detected in a significant amount within the biofilms. The substantial enrichment of biofilms with diatoms potentially suggests the implication of these microorganisms in the process of ennoblement. A mechanism is proposed in this paper describing the possible interactions of diatom community with SS in the studied ecosystem.

## 1. Introduction

Diatoms are microscopic, unicellular, autotrophic algae which are members of the heterokontic group of cells and are responsible for a major portion of the primary productivity, thus forming the base of the food chain in aquatic ecosystems. Based on their morphology, two types of diatoms are identified, centric with a radial symmetry and pennate with a bilateral symmetry. In general, the centric types are abundant in the water column, whereas the pennate ones are found in biofilms formed on natural or artificial surfaces. This is due to their ability to attach to substrata via extracellular polymeric substances (EPS) which are secreted through a structure known as a raphe [1]. Depending on the positioning and number of raphes, pennate diatoms are classified as monoraphid (one raphe), biraphid (two raphes, both present on one valve or one on each valve), and araphid (no raphe). Some diatom species attach to substrata with the help of structures such as stalks or pads [2]. Both centric and pennate types are often observed in each other's habitats. As centric types do not possess raphes, they are found entangled within the biofilm community. Both forms occur as single cells or as colonies in various shapes (filaments, ribbons, fans, zigzags, or even stellate). Within the biofilms, these diatoms play a similar role as their counterparts in the water column forming the base of the benthic food webs. Although the role of diatoms as primary producers in aquatic environments is of major importance, their presence on artificial man-made structures is a serious issue. Diatom fouling may, indeed, greatly affect the performance of metallic materials particularly used in structures located in marine and freshwater environments, resulting in huge economic loss. Diatom fouling on artificial substrata and strategies to prevent this process has been the subject of vast literature, especially in marine environments [3–13].

Stainless steels (SS) are among the most widely used materials in equipment having permanent or intermittent contacts with natural waters (seawater, freshwaters, estuaries, etc.). The exposure of SS to natural waters usually results in the increase (ennoblement) of the open circuit potential (OCP) which is largely attributed to the microbial activities within the biofilm [14–16]. The involvement of diatoms in the potential ennoblement is however not fully understood because diatom fouling communities and their role in microbiologically influenced corrosion (MIC) remain poorly documented (see review: [17] and references therein).

Diatoms are characterized by various growth forms described in terms of motility, colony, and extracellular mucilaginous matrix [18, 19]. Each growth form has significant ecological implications resulting in different community structures which depend on the age of the biofilm and the environmental conditions. In the context of biofouling, understanding the individual behavior of diatoms at solid substrata is important for the design of antifouling surfaces. Information on diatom colonization on various types of substrata and biofilm age is available [1, 20–23]. Most of these studies have been conducted in marine environments, while investigations in natural freshwaters remain poorly documented [17]. Moreover, there is limited information regarding diatom communities and their growth forms in biofilms formed after long-term exposures [24], that is, after several months. These features are with primary importance as an antifouling surface may be efficient for “early stage” biofilms but not as effective for “long-term” biofilms.

This study was conducted with the aim of generating information on the diatom fouling communities on SS substrata from a freshwater habitat after a long-term exposure (about six months). The fouling diatom community is grouped based on the growth forms and their ecological implications are discussed. The impact of diatom fouling on the stability of SS surfaces is also examined by monitoring the electrochemical response of the materials in situ during the natural exposure.

## 2. Experimental Procedures

### 2.1. Natural Exposure

Austenitic (AISI 304L and AISI 316L) and superaustenitic (254SMO) stainless steel (SS) samples (Outokumpu, Stainless AB, Finland) were used for this study. Their chemical compositions are given in Table 1. The SS surfaces were mechanically polished (both sides) with SiC papers of 600 and 1200 grain size to remove native oxides and other contaminants which may originate from material processing and to uniformize surface topography for all SS samples regardless of their type. After polishing, samples were rinsed with deionized water/ethanol (1 : 1, v/v) in a sonication bath (70 W, 40 kHz, Branson, USA) and dried under nitrogen gas flow. The natural exposure tests were conducted in the period of July to January by immersing SS samples in the Oise River (Verberie site located in the north of France, see Table 2 for more details) at a depth of about 1.50 m.

### 2.2. In Situ OCP Monitoring

During the natural exposure, the electrochemical behavior of SS samples was monitored continuously for about 40 days. For this purpose, the open circuit potential (OCP) of the SS electrodes was recorded in situ using a holder designed specifically for the experiment (see Figure  S1 in Supplementary Material available online at https://doi.org/10.1155/2017/5052646). It includes 20 SS samples and 5 reference electrodes (saturated calomel electrode (SCE)) placed at equal distances with respect to the SS working electrodes (i.e., one reference electrode for four SS samples). The SS samples were fixed to the holder at both sides, as shown in Figure  S1. At one side, the electric contacts between SS specimens and copper wires were embedded in an epoxy resin (Epofix, Struers, Denmark). At the other side, the samples were maintained by a Teflon holder. Data recording was performed using a multiplexer (Agilent 34970A) equipped with BenchLink DataLogger® software, which allows the OCP values to be measured every 10 min.

### 2.3. Surface Characterization

After natural exposure, the SS surfaces were rinsed with distilled water and dried under gentle nitrogen gas flow. Microscopic observations of SS surfaces were carried out without any preliminary chemical treatment using an environmental scanning electron microscope (ESEM, FEI Quanta FEC 250, FEI, USA). ESEM images were recorded on, at least, three different samples for each SS type. Diatom species identification was performed using micrographs, on the basis of flora reference [2, 25–28].

The surface composition of SS samples was determined by means of energy-dispersive X-ray spectroscopy (EDX) analyses performed using PGT IMIX-PTS apparatus (FEI, USA).

To provide a rough estimate of biofilm thicknesses, after natural exposure, SS samples were rinsed with deionized water, dried with nitrogen gas flow, and coated with epoxy resin to set the SS/biofilm interface immobilized. The samples were then cross-sectioned, polished, and thoroughly rinsed for ESEM observations.

### 2.4. Detection of Hydrogen Peroxide (H_2_O_2_) within the Biofilm

The presence of H_2_O_2_ within biofilms formed on SS surfaces after an exposure of about 1, 3, and 6 months in the natural medium was checked using analytical test papers (Merck no. 1.10011.0001). This method provides semiquantitative information regarding the amount of H_2_O_2_ present within the biofilm for concentrations ranging from 1 to 100 mg L^−1^.

## 3. Results

### 3.1. Biofilm Formation on SS Surfaces

**Figure 1:**
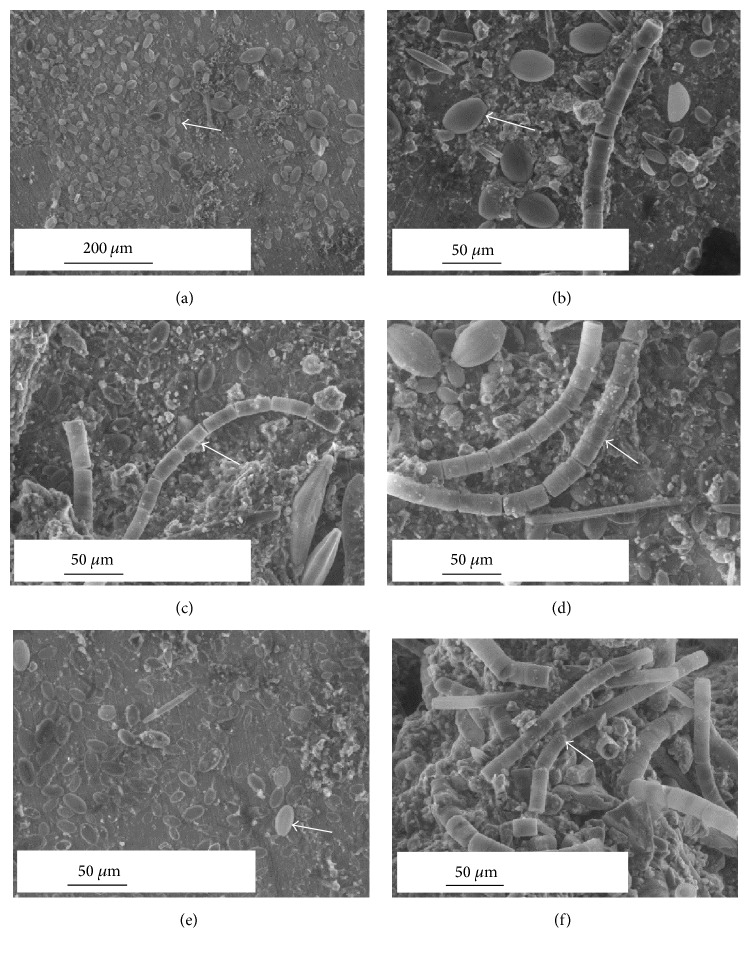
Representative ESEM micrographs showing diatom colonization on different types of SS (304L, 316L, 254SMO) exposed for six months in the natural river. Arrows in plates indicate* Cocconeis placentula* dominated community on 304L SS ((a), (b)),* Melosira varians* and other pennate diatoms dominated on 316L SS ((c), (d)),* C. placentula* dominated community (e), and* M. varians* dominated community on 254SMO SS (f). Horizontal line indicates the scale bar (50 and 200 *µ*m).

**Figure 2:**
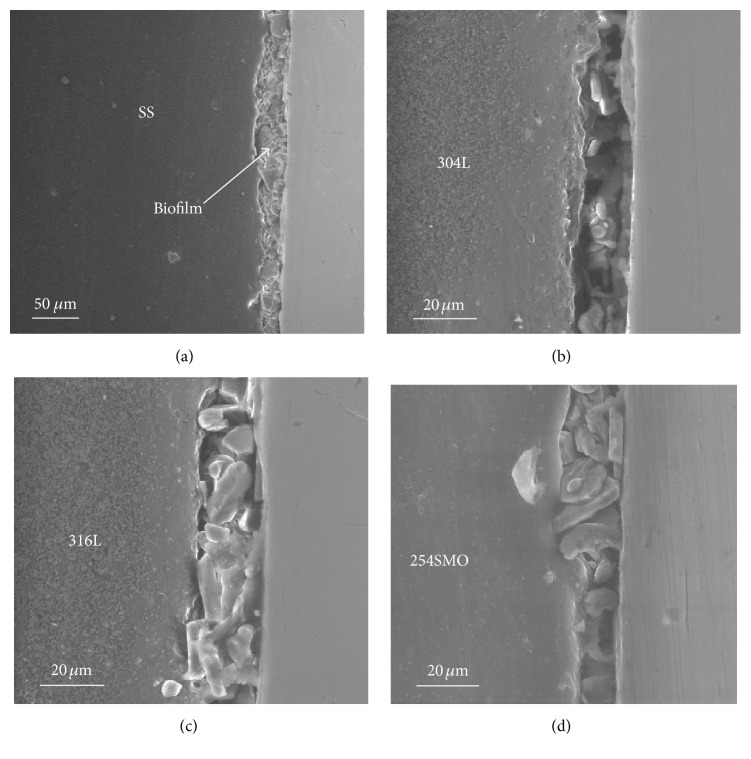
Representative ESEM images recorded on SS samples showing the thickness of biofilms formed on ((a), (b)) 304L (at different magnitudes), (c) 316L, and (d) 254SMO SS.

Typical ESEM micrographs of SS surfaces after about a six-month exposure in the natural river revealed the presence of heterogeneous biofilms (Figure 1). The enrichment of biofilms with diatom community is obvious in all the micrographs, regardless of the SS type (Figure 1). The morphological heterogeneous nature of biofilms was reflected in the degree of coverage of the SS surfaces. In some cases, the biofilm did not cover the SS surface totally, as evidenced by the stripes due to polishing, and, in other cases, a thick and dense film was observed which almost totally covered the SS surface (Figures 1(c), 1(d), and 1(f)). The biofilm thicknesses estimated in the dried state are around 20 *μ*m but may significantly vary along the SS/biofilm interface, as shown in Figure 2. However, it must be kept in mind that drying causes a significant loss of water content of the biofilm and, probably, morphological changes, leading to an underestimation of its thickness. Noticeable variations of the biofilm thickness were observed on the different SS samples, but no clear trend can be established as a function of the SS type (Figures 2(b)–2(d)).

Regarding the chemical composition of biofilms, a particular interest was dedicated to the presence of H_2_O_2_, owing to its ability to alter the stability of SS (for more details, see Section 4). It is worth remembering that the analytical paper test used for H_2_O_2_ is usable for H_2_O_2_ concentrations ranging from 1 to 100 mg L^−1^ (Figure  S2 in Supplementary Material). As a control, the analytical test paper wetted in the natural river remained uncolored, indicating that the amount of H_2_O_2_ was below the detection limit in the ambient freshwater. In contrast, all the tests conducted on the biofilms were positive and reproducible, showing a concentration in the order of 100 mg L^−1^ at different exposure times (1, 3, and 6 months), thus confirming the presence of a significant concentration of H_2_O_2_ within the biofilms (Figure  S2). This result is in agreement with previous studies conducted both in seawater [29] and in natural rivers [30].

**Figure 3:**
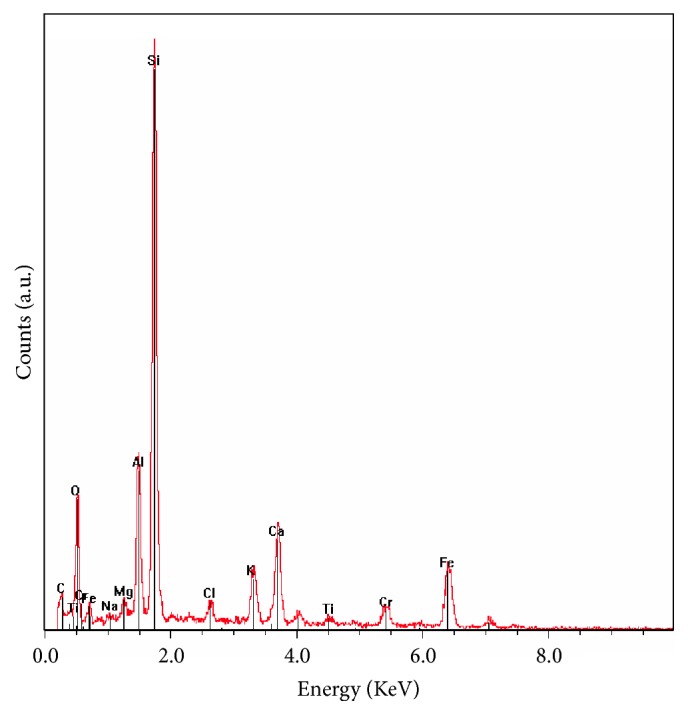
Representative EDS pattern recorded on SS sample after a six-month exposure in the natural river.

Further surface characterizations were performed on SS surfaces after their immersion in the natural medium using EDX analyses. A representative spectrum recorded on a SS sample is shown in Figure 3. Despite the heterogeneous nature of biofilms, EDX spectra showed similar pattern, revealing the presence of elements originating from the SS substratum, including bulk and oxide layer (Fe, Cr, Ni, O, etc.), salts, and organic compounds. More importantly, EDX spectrum revealed the dominating presence of silicon (Si), present in a small amount in the SS bulk (Table 1), which mainly originates from the diatom frustules (Figure 3). The intensity of Si peak varied slightly from one analyzed zone to another and from one sample to another (data not shown), but the trend remained almost the same with a significant amount of silicon in the presence of diatoms.

### 3.2. Fouling Diatom Community

**Figure 4:**
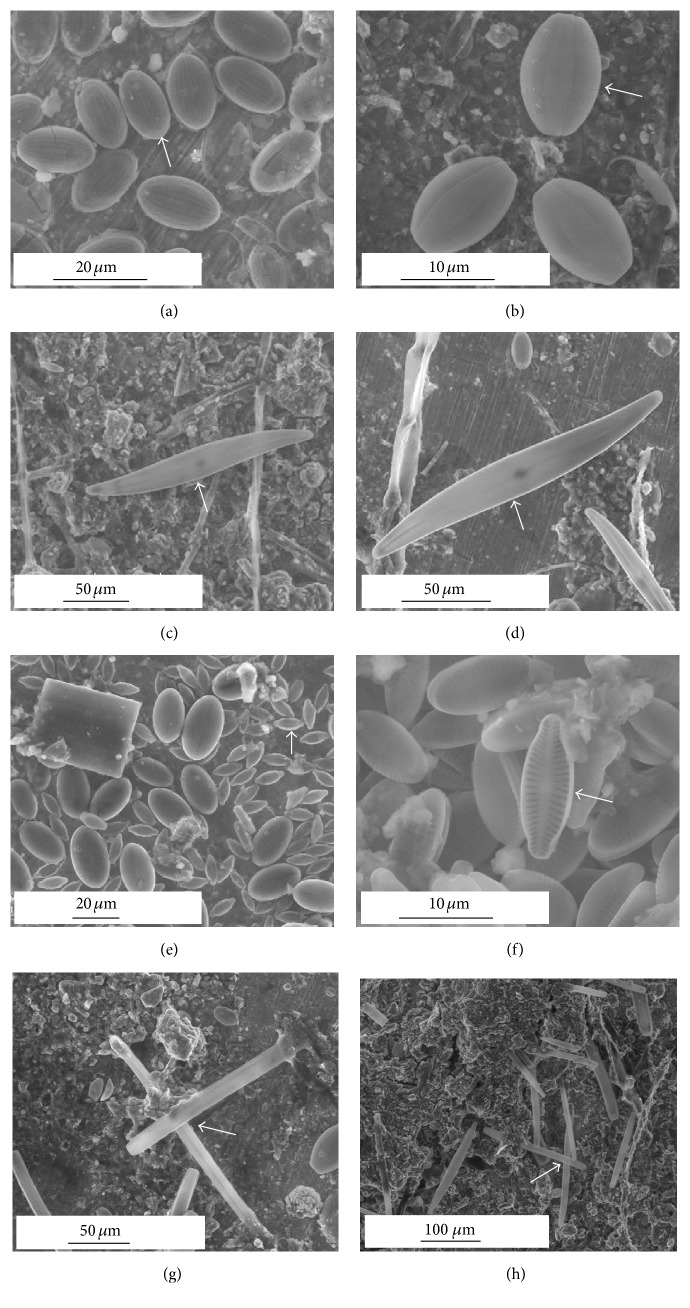
ESEM micrographs showing the diatom species composition present on the SS specimens after a six-month exposure period in the natural river: arrows in plates ((a) to (f)) indicate* Cocconeis placentula *(a),* Amphora ovalis *(b),* Gyrosigma* sp. ((c), (d)),* Placoneis elginensis *along with other pennate diatoms ((e), (f)), and* Nitzschia sigmoidea *are embedded in the biofilm matrix ((g), (h)). Horizontal line indicates the scale bar (10, 20, 50, and 100 *µ*m).

**Figure 5:**
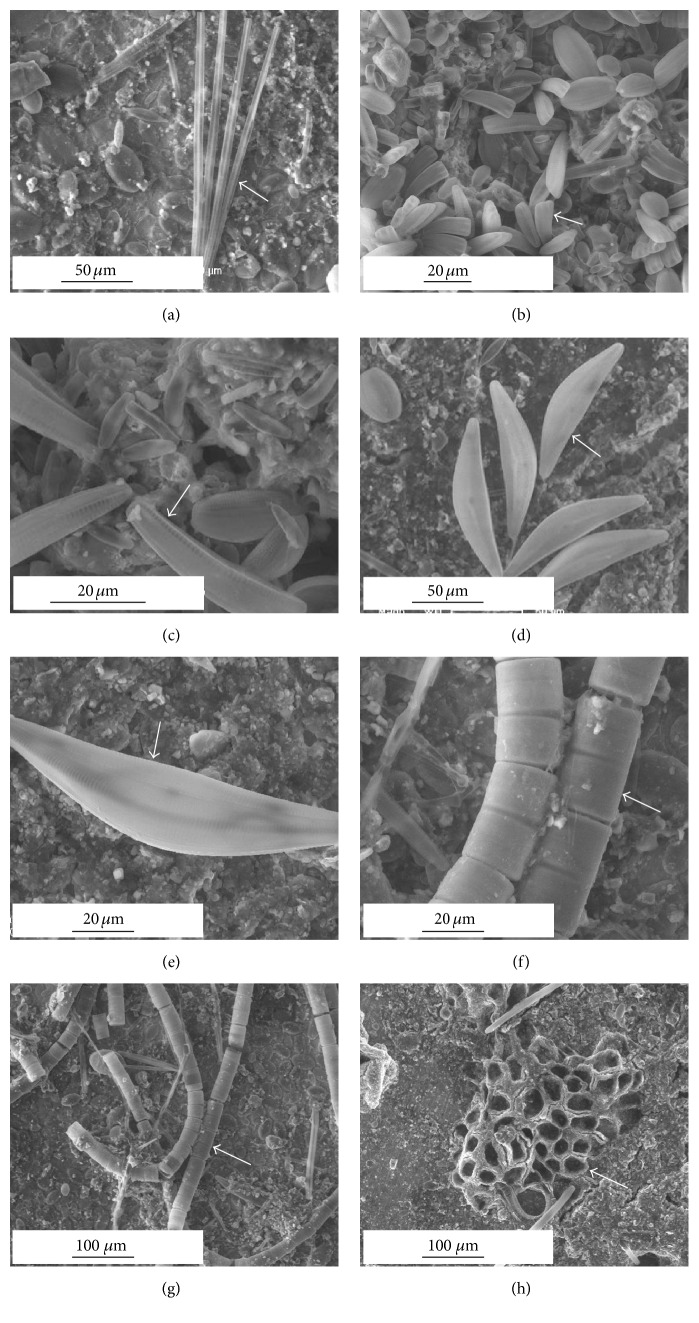
ESEM micrographs showing the composition of diatom species present on the SS specimens after a six-month exposure period in the natural river: arrows in plates ((a) to (h)) indicate the presence of* Synedra ulna* (a),* Rhoicosphenia abbreviata *along with other pennate diatoms ((b), (c))*, Cymbella *sp. ((d), (e)), chains of* Melosira varians* ((f), (g)), and* Spongia* (h). Horizontal line indicates the scale bar (20, 50, and 100 *µ*m).

A thorough examination of ESEM images allowed the identification of dominant diatom growth forms and species to be made. Nine diatom species were encountered on the SS surfaces: one centric and eight pennate species (Table 3). The pennate diatoms were attached to the substratum and included* Cocconeis placentula*,* Amphora ovalis*,* Gyrosigma* sp.,* Placoneis elginensis*, and* Nitzschia sigmoidea* (Figures 4(a)–4(h)). Another rosette type pennate diatom,* Synedra ulna*, was observed with individual cells held together by small mucilage pads produced at the poles of the valves leading to the formation of colonies due to common attachment on the substratum (Figure 5(a)).* Rhoicosphenia abbreviata* occurred as a stalked pennate diatom, whereas* Cymbella* sp. was directly attached to the substratum (Figures 5(b) and 5(c)). The only centric form,* Melosira varians*, was encountered in high numbers as a filamentous and unattached species (Figures 5(f) and 5(g)).

A detailed analysis of ESEM images reveals the influence of the SS type on the composition of the diatom communities (Table 4). It appears, indeed, that pennate diatoms,* C. placentula* and* A. coffeaeformis*, and the centric diatom,* M. varians* (Table 4 and Figure 1), were the abundant forms representing the fouling diatom community on all three SS types. However, while both the pennate and centric species were dominant on 316L and 254SMO types, only pennate species were dominant on 304L. Among 316L and 254SMO,* M. varians* was dominant on the former substrata (Table 4 and Figure 1).

### 3.3. Evolution of Open Circuit Potential

**Figure 6:**
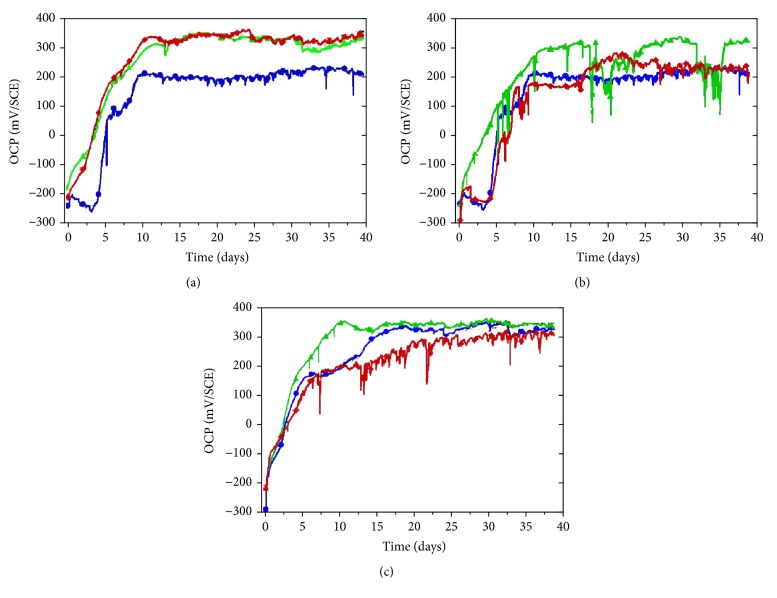
Typical OCP evolution recorded on different SS samples upon exposure in the natural river: (a) 304L and (b) 254SMO SS types.

One relevant way to evaluate the behavior of SS upon natural exposure is to record their open circuit potential (OCP). This method has the advantages to be noninvasive and to record events in situ, that is, during the colonization of microorganisms and the development of the biofilm. Typical evolutions of OCP recorded on the different SS types are given in Figure 6. Results revealed a significant increase of OCP towards positive values, higher than +200 mV/SCE after about ten days of immersion. The trends observed on 304L (Figure 6(a)), on 316L (Figure 6(b)), and on 254SMO (Figure 6(c)) samples were similar, and the potential fluctuations generally exhibited low amplitude (few mV). Moreover, the ennoblement either was immediate or occurred after some days (less than 5 days). In natural freshwaters, this delay, called “latency time,” has been already observed and seems to be mainly due to the variation of the water temperature which influences the biofilm development on SS surfaces and its microbial activities [30]. However, the phenomenon remains not well understood as many controversies regarding its origin are reported in the literature. This is indeed related to the complexity of natural rivers and the large variations from one location to another, in terms of composition and physicochemical parameters, which make any attempt of generalization difficult. Nevertheless, our results clearly support the hypothesis of a systematic OCP increase in natural rivers, sometimes contradicted (see [16] and references therein), independently of the SS composition (Table 1) and their microstructure (austenitic versus superaustenitic).

## 4. Discussion

It is obvious that diatoms are important in the biofouling community that develops on SS and other artificial substrata in natural freshwaters. In spite of its importance, diatom populations remain poorly documented compared to other microorganisms, namely, bacteria, especially in studies dealing with potential ennoblement [17, 30, 31]. In the present study, the immersion of SS substrata in the natural river for about six months led to the development of heterogeneous biofilms highly enriched with diatoms. This is the first report of long-term (i.e., several months) fouling diatom community on SS substrata from a freshwater ecosystem and its potential role in the electrochemical behavior of SS.

The dominance of pennate diatoms in the fouling film is a ubiquitous observation [9] due to their ability to attach to any surface. In previous studies, various growth forms of the attached diatom community have been described in terms of motility, colony form, and extracellular mucilaginous matrix form [32]. They are grouped into loosely attached motile forms, adnate (attaching with discs) and with short and long stalks. In the process of diatom fouling, a sequence is usually observed based on the appearance of the growth forms. Generally, raphid diatoms are among the earliest and most abundant primary colonizers of natural and artificial surfaces [32]. Cells adhere prostrate on the substratum, with the entire cell remaining close to the substratum surface [33]. These diatoms belong to the group of “tightly attached alga” including forms that grow oppressed to the substratum.* C. placentula* and* A. ovalis* belong to this group of diatoms. Furthermore, they are slow-moving solitary forms and are both epi- or endopsammic [18]. They have strong attachment capabilities [34], even under low light intensities [35], and tolerate darkness and anoxia for several days [36]. Also, heterotrophy is common among benthic diatoms [37]. These adaptations are important for prostrate, slow-moving cells subjected to frequent smothering or burial by other cells, detritus, and sediment [38].* C. placentula* is tolerant of moderate, but not severe, organic pollution and is a good indicator of eutrophication [39]. Members of these genera have been reported from toxic [33] and nontoxic [20, 21] surfaces.

The motile forms are followed by stalked diatoms which raise themselves from the surface and thus better utilize the vertical dimension. These nonmotile colonial forms can be subdivided according to the vertical expansion of the colonies over the substratum (Table 3). With respect to the genera encountered on the SS surfaces, in ascending order of uprightness are the linear chains (*Melosira*), arborescent forms (*Cymbella* and* Rhoicosphenia*), and fan-shaped colonies (*Synedra*).* Synedra* is known to possess high adhesive strength [40].* Rhoicosphenia* is an indicator of eutrophication and pollution [39].* Cymbella* is an asymmetrical biraphid diatom with cells growing predominately in benthic habitats and often producing mucilaginous stalks that are secreted through the apical pore field (Figures 5(d) and 5(e)). Members of these stalked diatom genera have been reported from toxic [24] and nontoxic [20, 21] surfaces.* M. varians* represents the nonmotile form with high light requirements [41, 42] and has been widely associated with eutrophic conditions (Figures 5(f) and 5(g)). These filamentous and unattached species are bottom-living centric forms that lack attachment mechanisms and maintain their position in flowing water through entanglement with species that attach to substrata with mucilaginous stalks [24]. In tropical estuarine waters, the centric species,* Melosira nummuloides*, and the pennate genera,* Navicula* and* Amphora*, have been reported to be the dominant forms encountered on SS surfaces [20, 43].

The success of each diatom species in the fouling community depends on the endurance of its growth form in response to the environmental conditions. The prostrate growth form and firm attachment via extracellular polymeric substances secreted by the raphe make* C. placentula* relatively resistant to scour and to selective grazers [44, 45]. The relative abundance of* C. placentula* was found to be high under strong current speed in rivers [46]. This species has the ability to complete its entire life cycle tightly attaching to the substratum surface, thereby avoiding shear forces that are experienced by other species that occupy a more elevated position from the surface [33]. The stalked diatoms have an advantage of acquiring nutrients from the surrounding waters due to their height as compared to the ones lying flat at the bottom. The first colonizers may be photoinhibited by the shading caused by the stalks and the cells at their tops and also nutrient limited [32]. The presence of the adnate forms in the six-month-old diatom community suggests that these cells were sturdy and hence were successful in maintaining their population. ESEM observations evidenced that diatoms may be present at different locations in the direction perpendicular to the SS surface plane. They were placed in close contact to “bare” SS surface, embedded within the biofilm, or also present on the top of the biofilm (Figures 1, 4, and 5).

Regarding the electrochemical behavior of SS upon natural exposure, the potential ennoblement was observed on all SS types (Figure 6). It was concomitant with the significant presence of H_2_O_2_ within biofilms. Earlier studies have identified H_2_O_2_ as one of the main intermediates of the oxygen reduction reaction within both marine and freshwater biofilms formed on SS substrata [29, 30, 47–49]. Furthermore, the nature of electrochemical processes involving H_2_O_2_ has been deeply investigated on SS in laboratory-controlled systems using natural sterilized seawater [50–53], artificial seawater [50–52], and artificial freshwater [30, 54, 55]. The desired concentration of H_2_O_2_ was obtained either through addition of a H_2_O_2_ solution or by in situ production through an enzyme-catalyzed reaction to mimic the microbial generation of H_2_O_2_ in biofilms. The biogenic formation of H_2_O_2_ is mainly controlled by enzyme-catalyzed reactions both for production (enzymes using O_2_ as electron acceptors) or for degradation (enzymes involved in processes against oxidative stress) [16]. The amount of H_2_O_2_ within biofilms is also affected by diffusion at the interface of SS/biofilm/solution which is generally influenced by the biofilm morphology, hydrodynamic effects, temperature changes, and so forth.

The large enrichment of the SS biofilms with diatoms may suggest a possible implication of these microalgae in the process of ennoblement. In the present study, this hypothesis is supported by the fact that OCP values measured after three and six months remain relatively high, that is, ranging from +200 to +360 mV/SCE. Indeed, an analysis of data reported in the literature [17] reveals the possibility of different mechanisms, involving diatoms: action through (i) photosynthetic activities and (ii) via bacteria-diatom interaction. The most relevant evidence regarding the photosynthetic activities relies on the light-dependent ennoblement [30, 48, 56, 57]. The photosynthetic metabolic activity, which leads to the production of O_2_ at the SS/biofilm interface, seems to induce only day-night variations that do not prevent the potential ennoblement. This may explain why ennoblement also occurred in dark conditions. In the present study, on some SS samples, periodic potential fluctuations were observed and may correspond to day/night cycles (Figure 6). However, the amplitudes of variation were lower compared to previous reports [30, 48]. These day/night potential variations can be attributed to enrichment/depletion cycles of oxygen at the SS/biofilm interface which can be indicated by considering the Nernst equation [58]. It predicts that production of oxygen would increase the electrode potential and vice versa. However, this process could not explain solely the potential ennoblement. The depletion of O_2_ at the SS/biofilm interface is connected to the production of H_2_O_2_, and other reactive oxygen species (ROS), through the aerobic microbial activities [16, 17], resulting in potential ennoblement. In a previous study, the depletion of O_2_ and enrichment of H_2_O_2_ were obtained at the vicinity of SS surface using a biomimetic biofilm [59]. Results showed a potential ennoblement similar to that observed in natural media which may be explained by the fact that H_2_O_2_ is a stronger oxidizer [60].

The most plausible explanation consists in an indirect action of diatoms on OCP by providing the photosynthetic metabolic product (O_2_) to other heterotrophic microorganisms present in the biofilm. This explanation was provided by Ishihara and Tsujikawa [61, 62] who examined the evolution of OCP values of SS upon a successive immersion in two different media. In the first media, the SS samples were immersed in natural seawater for several days in a way that OCP ennoblement did not significantly exceed values around +100 mV/SCE. The SS samples were then transferred to the second medium, a diatom-enriched solution in which ennoblement reached values near +400 mV/SCE. The authors showed that the latter medium (enriched with diatoms) did not lead, solely, to a significant increase of OCP, suggesting that both media are required to reproduce the ennoblement observed in natural conditions. These relevant findings suggest that the ennoblement is the result of a combined action of diatoms and bacteria. Diatoms, through their photosynthetic metabolic activities, provide O_2_ to heterotrophic bacteria, also present in the biofilm, to be used in the different aerobic metabolic processes, including biogenic formation of H_2_O_2_, as described above. According to the same authors, the ennoblement did not occur in the diatom-enriched medium because diatoms are not able to adhere to the SS surface without a previous surface colonization and adhesion of bacteria.

In our study, it appears that diatoms may be in a direct contact with SS surface (see Figures 1 and 4). The ability of diatoms to adhere to surfaces in the absence of bacteria has been also observed elsewhere [8]. However, it must be kept in mind that the latter study has been conducted in the laboratory under controlled conditions with absence or presence of bacteria. According to Wahl [63], in natural systems, the pattern of bacteria colonizing surfaces before diatoms is either due to their higher relative abundance in the immigrant pool [64] or because they facilitate diatom attachment [65]. Furthermore, it has been reported that the role of bacteria is variable wherein bacteria may facilitate algal attachment if the base material is hydrophilic or has no effect and even inhibit algal attachment if the base material is hydrophobic [64]. Therefore, the type of surface along with the type of bacterial community will influence the bacteria-diatom interactions in the natural environment which will in turn influence the process of ennoblement. Thus, the hypothesis of a combined action of diatoms and bacteria is promising and the question clearly deserves further investigation, as the mechanism of interaction between bacteria and diatoms, from the microbiological point of view, remains not fully understood [66, 67].

The complexity of this process is inherent in the multiplicity of parameters influencing the interaction between diatoms and bacteria especially at the SS/biofilm interface. Although other parameters related to the immersion conditions (temperature, location, etc.) are also relevant, our results provide a promising connection between ennoblement and diatom fouling and are particularly important in biofouling and related issues. From an ecology perspective, the interaction of diatoms with SS may be extended to other natural or artificial substrata to provide insights into the fate and behavior of diatoms in these specific conditions and to evaluate the impacts on the surrounding ecosystem.

## 5. Conclusion

In this article, a thorough analysis of diatom community is performed on biofilms formed on SS materials upon exposure in a natural freshwater ecosystem (Oise River). ESEM images revealed the dominance of prostrate growth forms such as* Cocconeis* and* Amphora* in the six-month-old biofilm (initial colonizers), regardless of the SS type, suggesting their strong attachment capability and tolerance to variable environmental conditions. Both pennate and centric species were dominant on 316L and 254SMO types, while only pennate species were dominant on 304L.

Regarding the behavior of SS upon natural exposure and the development of diatom-enriched biofilms, it appears that the ennoblement observed is due to biogenic production of H_2_O_2_ and potentially may involve diatoms, probably through a combined action with heterotrophic bacteria.

## Supplementary Material

Details of the holder used for natural exposure of SS and electrochemical monitoring; analytical paper test used for the detection of hydrogen peroxide.

## Figures and Tables

**Table 1:** Standard designations and chemical composition (wt.%) of stainless steel (SS) types used in this study.

AISI designation	ISO designation	wt.%								
		C	Si	Mn	Cr	Ni	Mo	N	Cu	Fe
304L	X2CrNi18-9	0.022	0.18	1.34	18.20	8.15	—	0.094	0.075	Balance
316L	X2CrNiMo17-11-2	0.019	0.48	1.65	16.8	10.61	2.58	0.041	0.36	Balance
254SMO	X1CrNiMoCuN20-18-17	0.020	0.80	1.00	20.00	18.00	6.25	0.020	0.75	Balance

**Table 2:** Mean parameters of freshwater (Verberie site, Oise river, France).

Parameter	Unit	Data^*∗*^	Standard deviation
pH	*—*	7.96	0.19
Temperature	°C	13.72	5.87
Conductivity at 25°C	*μ*S·cm^−1^	617.64	59.38
Turbidity	NTU	21.92	21.84
Total suspended matter	mg·L^−1^	27.88	26.95
COD	mg·L^−1^	15.67	6.82
Redox potential	mV	160.17	26.57
BOD5 at 20°C	mg·L^−1^	2.08	0.83
Dissolved oxygen	mg·L^−1^	10.36	1.55
Chloride [Cl^−^]	mg·L^−1^	29.00	7.38
Bicarbonate [HCO_3_^−^]	mg·L^−1^	280.17	25.90
Carbonate [CO_3_^2−^]	mg·L^−1^	2.62	0.49
Phosphate [PO_4_^3−^]	mg·L^−1^	0.5	*—*
Nitrate [NO_3_^−^]	mg·L^−1^	17.14	3.71
Nitrite [NO_2_^−^]	mg·L^−1^	0	0
Sulfate [SO_4_^2−^]	mg·L^−1^	38.17	6.18
Na	mg·L^−1^	17.00	5.87
Mg	mg·L^−1^	7.50	1.38
Silica (SiO_2_)	mg·L^−1^	11.13	0.99
Chlorophyll a	*µ*g·L^−1^	4.25	4.10

^*∗*^Data based on three-year average from the French Water Agency for Seine-Normandy Water (http://www.eau-seine-normandie.fr).

**Table 3:** Taxonomic list of diatoms present on the stainless steel after immersion in the natural freshwater for about six months.

Class	Type	Species	Solitary/colonial	Form	Raphe
Coscinodiscophyceae	Centric	*Melosira varians* Agardh (1827)	Chain colony	Floating	Araphid
Bacillariophyceae	Pennate	*Amphora ovalis* Kutzing (1844)	Solitary cell	Adnate	Biraphid
Bacillariophyceae	Pennate	*Cocconeis placentula* Ehrenberg (1838)	Solitary cell	Adnate	Monoraphid
Bacillariophyceae	Pennate	*Cymbella* sp. Agardh	Arbuscular colony	Mucilage stalk-upright	Biraphid
Bacillariophyceae	Pennate	*Gyrosigma* sp. Hassall	Solitary cell	Motile	Biraphid
Bacillariophyceae	Pennate	*Nitzschia sigmoidea* W. Smith (1853)	Solitary cell	Motile	Biraphid
Bacillariophyceae	Pennate	*Placoneis elginensis* (Gregory) Cox (1987)	Solitary cell	Motile	Biraphid
Bacillariophyceae	Pennate	*Rhoicosphenia abbreviata* (Agardh)	Arbuscular colony	Mucilage stalk-upright	Biraphid
Fragilariophyceae	Pennate	*Synedra ulna* Ehrenberg (1836)	Rosette colony	Mucilage stalk-upright	Araphid

**Table 4:** Relative abundance of diatom species on the different SS types (“−” = absent; “+” = low; “++” = moderate; “+++” = high; “++++” = very high).

Species	304L	316L	254SMO
*Melosira varians*	++	+++	+++
*Amphora ovalis*	+++	++	++
*Cocconeis placentula*	++++	++++	++++
*Cymbella* sp.	+	+	+
*Gyrosigma* sp.	+	+	+
*Nitzschia sigmoidea*	++	+	+
*Placoneis elginensis*	−	+	+
*Rhoicosphenia abbreviata* (Agardh)	+	+	−
*Synedra ulna*	−	+	+
