# Supplementary material for: Diagnosis of the Diatom Community upon Biofilm Development on Stainless Steels in Natural Freshwater
Source: Scanning. 2017 May 25;2017:5052646. doi: 10.1155/2017/5052646 (PMC5662069; doi:10.1155/2017/5052646)
Supplement: Supplementary file 1 — Details of the holder used for natural exposure of SS and electrochemical monitoring; analytical paper test used for the detection of hydrogen peroxide. [file 5052646.f1.docx]

Diagnosis of the diatom community upon biofilm development on stainless steels in natural freshwater

# Caroline Richard ^a,*^, Smita Mitbavkar ^b^, Jessem Landoulsi ^c, d, *^

***Supplementary materials***

**Figure S1.** Sample holder used for open circuit potential (OCP) monitoring during the natural exposure of stainless steel samples.

**Figure S2.** Analytical paper test (Merck N° 1.10011.0001) used for the detection of hydrogen peroxide (H_2_O_2_) within biofilms. Test results obtained after a contact with (B) the natural freshwater or with (C) biofilms formed on SS samples after an immersion of about one and six months.
